# Biomarkers of Brain Damage: S100B and NSE Concentrations in Cerebrospinal Fluid—A Normative Study

**DOI:** 10.1155/2015/379071

**Published:** 2015-09-01

**Authors:** Lenka Hajduková, Ondřej Sobek, Darina Prchalová, Zuzana Bílková, Martina Koudelková, Jiřina Lukášková, Inka Matuchová

**Affiliations:** ^1^Laboratory for CSF and Neuroimmunology, Topelex Ltd., 190 00 Prague, Czech Republic; ^2^Department of Neurology, The Military University Hospital Prague, 169 02 Prague, Czech Republic; ^3^Department of Biology and Medical Genetics, Charles University 2nd Faculty of Medicine and University Hospital Motol, 150 06 Prague, Czech Republic; ^4^Department of Biochemistry and Microbiology, The Institute of Chemical Technology, 166 28 Prague, Czech Republic

## Abstract

NSE and S100B belong among the so-called structural proteins of the central nervous system (CNS). Lately, this group of structural proteins has been profusely used as specific biomarkers of CNS tissue damage. So far, the majority of the research papers have focused predominantly on the concentrations of these proteins in blood in relation to CNS damage of various origins. Considering the close anatomic and functional relationship between the brain or spinal cord and cerebrospinal fluid (CSF), in case of a CNS injury, a rapid and pronounced increase of the concentrations of structural proteins specifically in CSF takes place. This study inquires into the physiological concentrations of NSE and S100B proteins in CSF, carried out on a sufficiently large group of 601 patients. The detected values can be used for determination of a normal reference range in CSF in a clinical laboratory diagnostics.

## 1. Introduction

Approximately from the 80s, there has been a notable increase of interest in structural proteins of the central nervous system (CNS), including S100B and NSE as biomarkers of CNS tissue damage [1].

### 1.1. Protein S100B

The exact term is S100 calcium binding protein B or S100B. This protein is the first identified member of the S100 protein multigenic family and participates in an extracellular and intracellular regulation of a cellular calcium metabolism [2]. Protein S100B, which is a major subordinate unit in mammals, is located mostly in glial cells of the central (predominantly astrocytes) and peripheral nervous system, but also in chondrocytes, melanocytes, and adipocytes [3].

S100B might have either a trophic or toxic effect depending on the local concentration. In a low nanomolar physiological concentration it seems to have a neurotrophic effect, it stimulates the growth of neurons, and it increases their survival during development and also during an injury [4–6]. On the other hand, higher concentrations of this protein might be toxic and evoke cell death. Generally, S100B acts like a damage-associated molecular pattern (DAMP), which is released from damaged or activated cells under conditions of cell stress [7]. Furthermore, a more complex role of S100B in the inflammatory processes has been described [8–11].

### 1.2. Neuron-Specific Enolase (NSE)

It is a dimer formed in neurons with subordinate units *α*-*γ* or *γ*-*γ*, which belongs to the group of hydrolytic enzymes. NSE is an isoenzyme of enolase (2-phospho-D-glycerate hydrolase), which catalyzes the transition of 2-phosphoglycerate into phosphoenolpyruvate [12]. It is present in tissues of neuroectodermal origin. In a small amount, NSE is present in erythrocytes, blood platelets, plasmatic cells, lymphocytes, capillary walls, and myoepithelial cells, which explains its physiologically low concentrations in blood [13, 14].

In case of a CNS injury, accompanied by a nervous tissue and cellular damage, these structural proteins are released from cells, and their concentrations increase extracellularly—including CSF and blood. In these consequences these proteins could be called “biomarkers of brain damage” [1, 15–18].

Considerable number of research papers, engaged in monitoring the concentrations of NSE and S100B protein after a traumatic brain injury (TBI), was published [15, 17, 19–23].

The concrete value and dynamics of the concentrations of S100B protein and NSE also play an important role in the prediction of the outcome of patients who had brain ischemia [15, 23–28] or bleeding [15, 29–31] or after they underwent a cardiac surgery [32, 33] or a neurosurgery [34, 35].

Proteins S100B and NSE are also produced by some tumorous cells of neuroectodermal origin [36, 37].

Furthermore, structural proteins of CNS, including NSE and S100B protein, were researched in relation to prion diseases of CNS [38].

Major part of the research studies focuses on monitoring the concentrations of these biomarkers in blood. Sporadic studies that address the determination of their physiological concentrations in CSF are carried out on relatively small groups of patients [39].

Considering not only the close anatomic relationship between the brain or spinal cord and CSF, but also the small volume of a CSF reservoir, when CNS tissue is damaged, a rapid and pronounced increase of the concentrations of structural proteins specifically in CSF takes place.

In our opinion, a substantial study of the physiological concentrations of NSE and S100B protein in CSF, carried out on a sufficiently large group of patients, is missing.

In the Laboratory for CSF and Neuroimmunology, Prague, there are approximately 5000 CSF samples analyzed per year. Biochemical analysis (CSF total protein, glucose, lactate, and albumin), CSF cytological analysis (cell count and qualitative cytology), immunological analysis (CSF immunoglobulins IgG, IgM, IgA, isoelectric focusing of immunoglobulins and free light chains kappa and lambda, inflammatory markers including IL 1, IL 6, IL 8, and IL 10, autoantibodies including anti-AQP4, Yo, Hu, Ri, NMDA, AMPA, GABA, VGKC, and Gangliosides), microbiological CSF analysis (PCR and antibodies against neurotropic microbial agents, including Borrelia Burgdorferi s.l., Treponema Pallidum, Herpes viruses, TBE virus), and determination of structural proteins of CNS, namely, S100B, NSE are performed in this laboratory workplace.

## 2. Materials and Methods

We were able to compile a large enough investigated group of approximately 600 patients, on which we determined sufficiently reliable range of normal values of NSE and S100B in CSF.

### 2.1. Investigated Group

From the group of altogether 28.394 patients whose CSF was obtained and analyzed for diagnostic purposes within the years 2008–2014 (suspected inflammatory, vascular, degenerative, or traumatic impairment of CNS), a file of 601 patients was selected. There were no pathological findings in these patients as well as no clinical or CT/MRI signs of CNS tissue damage. Biochemical, cytological, and immunological values in CSF were normal.

### 2.2. Inclusion Criteria

 Total cell count in CSF <= 4/*μ*L.
 Normal cytological finding: 60–80% of lymphocytes, no plasma cells, monocytes without signs of activation, no phagocytosis, no granulocytes, no atypical or tumorous cells.
 Total CSF protein <= 0.4 g/L.
 Lactate in CSF <= 2 mmol/L.
 Glucose in CSF >= 2.2 mmol/L AND <= 4.2 mmol/L.
 Coefficient of energy balance (CEB) in normal range (>27) [40, 41].
 Negative finding of oligoclonal bands (OCB) in CSF on isoelectric focusing (IEF) of IgG (pattern I-normal finding).
 IL 6 in CSF < 18 pg/mL.

Patients signed an informed consent with a further scientific use of their CSF samples.

### 2.3. Analytical Method

For determination of the concentrations of S100B and NSE in CSF, the sensitive electrochemiluminescence immunoassay (ECLIA, Roche Diagnostics) on Elecsys 2010 analyzer was used.

The method is based on the use of a ruthenium-complex and tripropylamine (TPA). The chemiluminescence reaction for the detection of the reaction complex is initiated by applying a voltage to the sample solution resulting in a precisely controlled reaction.

The standard kits for in vitro diagnostics, NSE catalogue number: 12133113 and S100B catalogue number: 03175243 by Roche Diagnostics GmbH, Sandhofer Strasse 116, D-68305 Mannheim, were used [42].

Interlaboratory comparisons of measurements of the concentrations of S100B and NSE were done in the frame of EQA (External Quality Assessment) organized by SEKK, Pardubice, Czech Republic.

The investigated group was divided into two groups of men and women to determine sex dependency and then into two age groups (20–59 and over 60 years) to establish age consequences.

The obtained data were statistically analyzed and scatter diagrams were created using program MS Excel. We estimated the reference limit as the 2.5th and the 97.5th percentiles for sex and age dependency.

## 3. Results and Discussion

See Table 1 and Figures 1, 2, 3, 4, 5, 6, 7, 8, 9, 10, 11, and 12.

### 3.1. Sex and Age Dependence

In the investigated group it was possible to observe similar tendencies in relation to age, as it had already been observed in previously published studies [39, 43, 44], that is, increasing concentrations of structural proteins of the CNS with age. In relation to sex, a higher concentration of S100B protein in CSF was observed in women, and, on the contrary, a higher concentration of NSE in CSF was observed in men.

Explanation of different allocation of both proteins, based on sex, could be most likely found in the dissimilar physiological tasks of each structural protein, eventually in their different distribution within the frame of the CNS—the source of S100B protein is astroglial cells above all, whereas NSE comes from the cellular body of neurons for the most part.

## 4. Conclusion

NSE and S100B are considered as quite established and specific markers of the CNS tissue damage. So far, clinical-laboratory interpretations mostly relied on studies based on monitoring of these structural proteins in blood.

Nonetheless, besides taking into account the close anatomic relationship between the brain or spinal cord and CSF, and also the small volume of a physiological CSF reservoir, in case of a CNS injury, the most sensitive and specific compartment accessible for a routine laboratory analysis is CSF.

With regard to the intercompartmental dynamics of a protein transportation from CNS to CSF [45], after a brain injury, a rapid and pronounced increase of the concentrations of the structural proteins particularly in CSF takes place.

This study evaluated the physiological concentrations of NSE and S100B protein in CSF, carried out on a sufficiently large group of 601 patients. Correlations of the concentrations of S100B and NSE depending upon age and sex were established as well.

The values detected in this study can be used for stating the normal reference range in CSF in a clinical laboratory diagnostics.

Reliable reference ranges of S100B and NSE are necessary for a clinical interpretation of laboratory findings in CSF in patients with suspected damage of CNS tissue. Preference of determination of concentrations of these proteins in CSF, to their determination in blood, is meaningful especially in a more discrete impairment of CNS (in terms of degenerative or vascular etiology, in traumatic or compressive impairment of brain and spinal cord, etc.). Into a large group of diseases, with a possible tissue damage of CNS, where CSF diagnostics is preferred, belong neuroinflammations, both neuroinfections and autoimmune processes (MS, NMO, ADEM, etc.).

Laboratory analysis used in this study is based on an established and widely accessible ECLIA methodology used in a routine laboratory practice. Presently, there are lots of regular external quality control assessments (EQA) for S100B and NSE available, so that interlaboratory comparability of results is guaranteed. Introduction of these new parameters into the CSF diagnostics brings, in the authors' opinion, a new and potent diagnostic tool for a detection of a CNS tissue damage, which is both independent of and complementary to neuroimaging methods like CT or MRI.

The precise determination of the presence and degree of a structural damage of CNS has a clinical significance for both prognosis of a patient's outcome and a choice of therapeutic approaches.

## Figures and Tables

**Figure 1:**
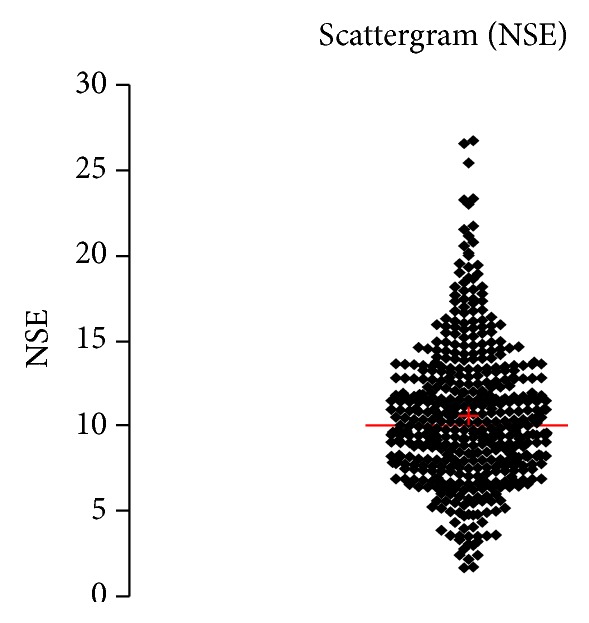
Concentration of NSE (*μ*g/L) in an age group of patients 20–59 years, indiscriminately of sex.

**Figure 2:**
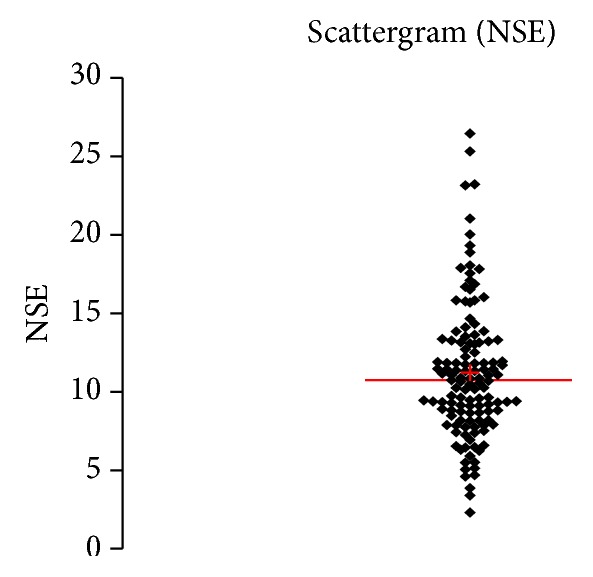
Concentration of NSE (*μ*g/L) in an age group of patients 20–59 years, men.

**Figure 3:**
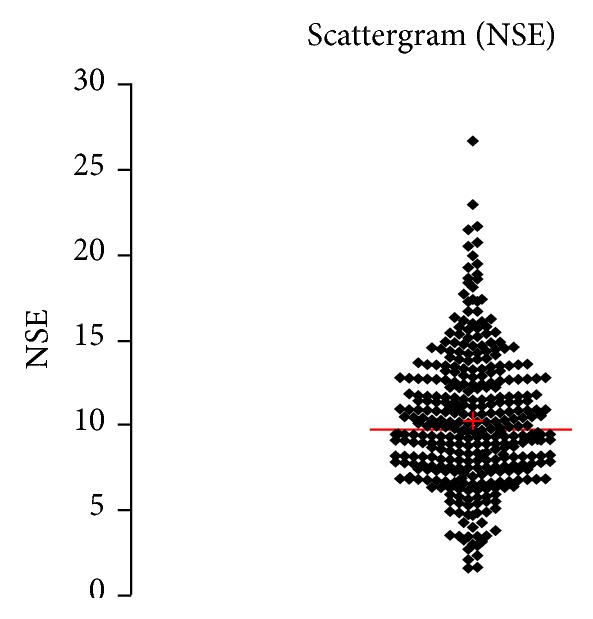
Concentration of NSE (*μ*g/L) in an age group of patients 20–59 years, women.

**Figure 4:**
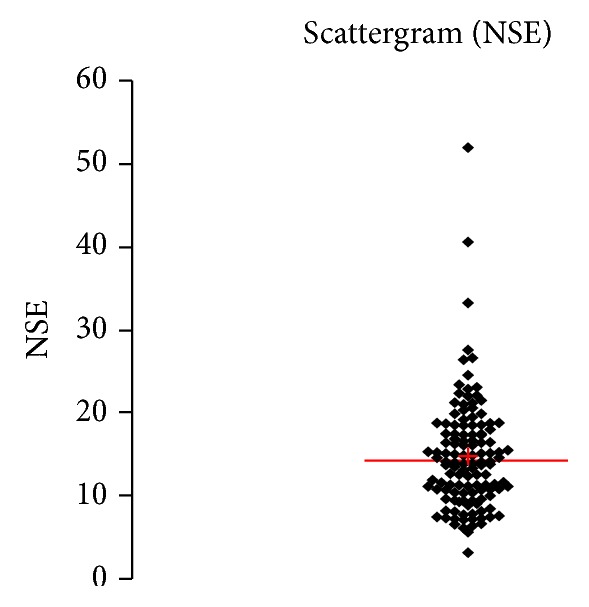
Concentration of NSE (*μ*g/L) in an age group of patients above 60 years, indiscriminately of sex.

**Figure 5:**
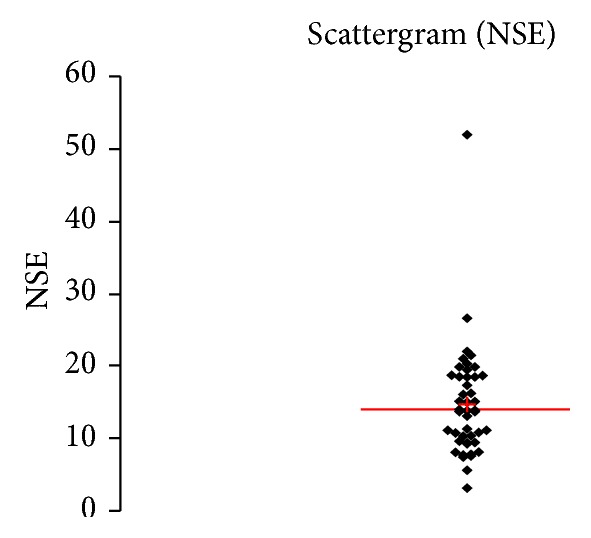
Concentration of NSE (*μ*g/L) in an age group of patients above 60 years, men.

**Figure 6:**
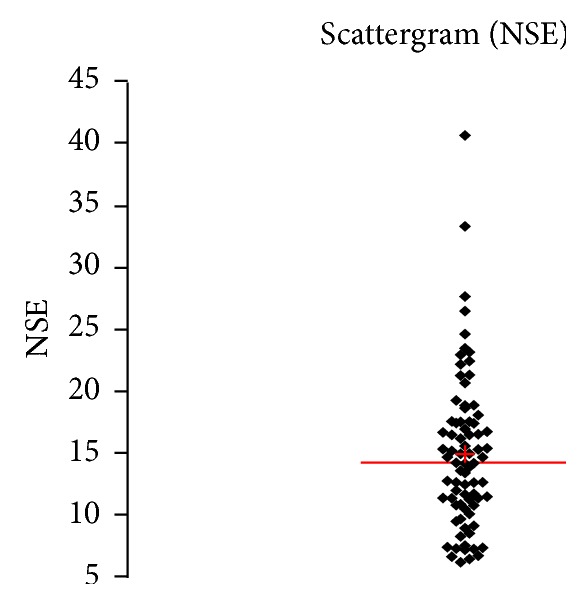
Concentration of NSE (*μ*g/L) in an age group of patients above 60 years, women.

**Figure 7:**
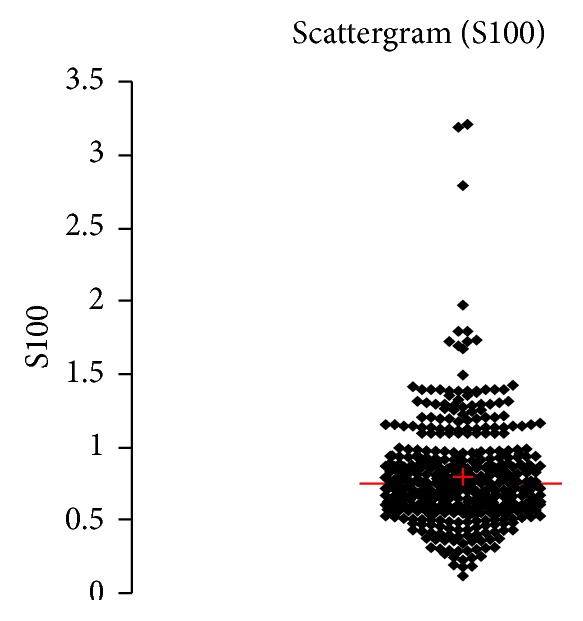
Concentration of S100B (*μ*g/L) in an age group of patients 20–59 years, indiscriminately of sex.

**Figure 8:**
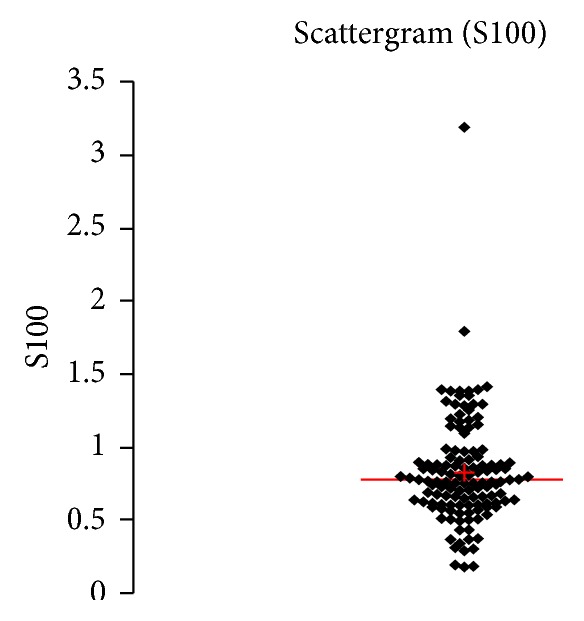
Concentration of S100B (*μ*g/L) in an age group of patients 20–59 years, men.

**Figure 9:**
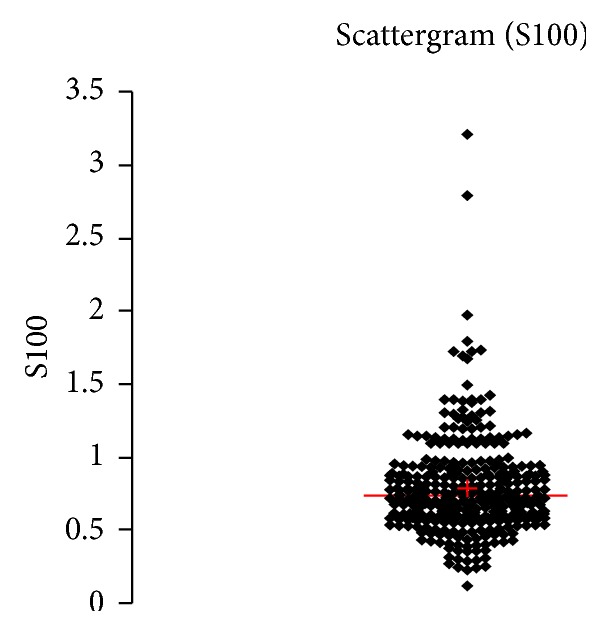
Concentration of S100B (*μ*g/L) in an age group of patients 20–59 years, women.

**Figure 10:**
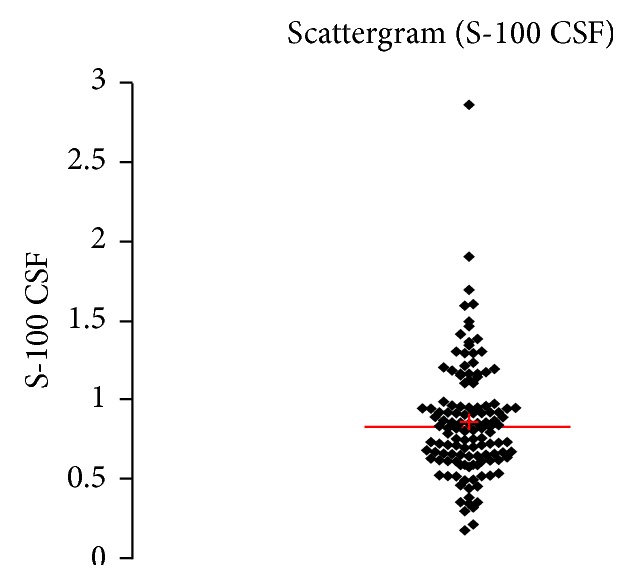
Concentration of S100B (*μ*g/L) in an age group of patients above 60 years, indiscriminately of sex.

**Figure 11:**
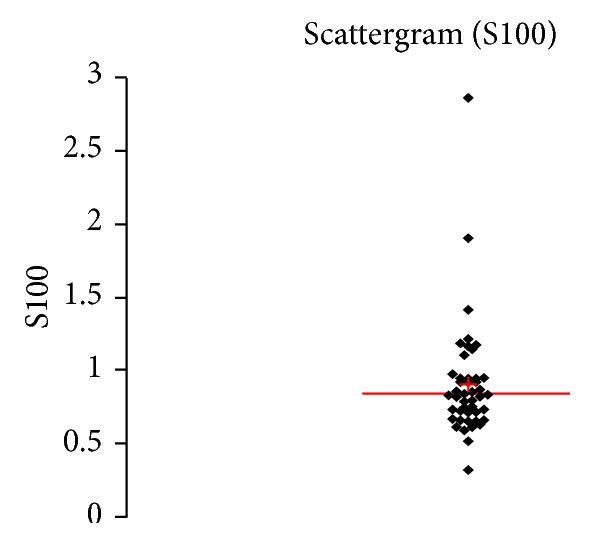
Concentration of S100B (*μ*g/L) in an age group of patients above 60 years, men.

**Figure 12:**
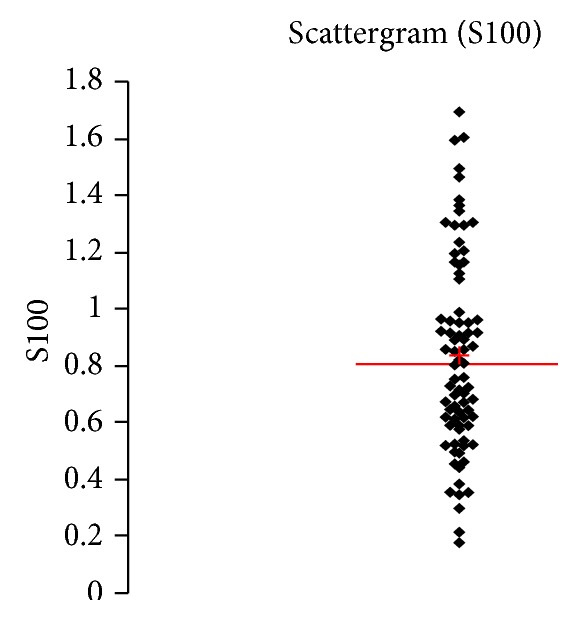
Concentration of S100B (*μ*g/L) in an age group of patients above 60 years, women.

**(a):** 

Percentile 2.5	Percentile 97.5
Whole group, *n* = 601	
0.304	1.600

Men, *n* = 171	
0.309	1.420

Women, *n* = 430	
0.303	1.600

Age 20–59 years, *n* = 477	
0.303	1.437

Age > 60 years, *n* = 124	
0.326	1.609

**(b):** 

Percentile 2.5	Percentile 97.5
Whole group, *n* = 601	
3.50	22.98

Men, *n* = 171	
4.67	23.29

Women, *n* = 430	
3.47	22.28

Age 20–59 years, *n* = 477	
3.47	19.98

Age > 60 years, *n* = 124	
6.46	27.63

The reference intervals were calculated as 2.5th and 97.5th percentiles.

## Abbreviations

CNS: Central nervous system
CSF: Cerebrospinal fluid
NSE: Neuron-specific enolase
S100B: S100B protein. Synonyms: S100 calcium binding protein B
DAMP: Damage-associated molecular pattern
TBI: Traumatic brain injury
AQP4: Aquaporin 4
Anti-Yo: Anti-Yo antibodies. Synonyms: type 1 Purkinje cell cytoplasmic autoantibodies (PCA-1).
Anti-Hu: Anti-Hu antibodies. Synonyms: anti-neuronal nuclear antibody type 1 (ANNA-1)
Anti-Ri: Anti-Ri nuclear antibody type 2 (ANNA-2)
NMDA: (N-Methyl-D-aspartate) receptor
AMPA: (*α*-Amino-3-hydroxy-5-methyl-4-isoxazolepropionic acid) receptor
GABA: (Gamma aminobutyric acid) receptor
VGKC: Voltage-gated potassium channel
IEF: Isoelectric focusing
CEB: Coefficient of energy balance
ECLIA: Electrochemiluminescence immunoassay
EQA: External quality assessment
MS: Multiple sclerosis
NMO: Neuromyelitis optica
ADEM: Acute disseminated encephalomyelitis
CT: Computed tomography
MRI: Magnetic resonance imaging.
